# Identifying Priority Geographic Locations for Diabetes Self-Management Education and Support Services in the Appalachian Region

**DOI:** 10.5888/pcd21.230297

**Published:** 2024-04-25

**Authors:** Jacob T. Wittman, Dayna S. Alexander, Melissa Bing, Robert Montierth, Hui Xie, Stephen R. Benoit, Kai McKeever Bullard

**Affiliations:** 1 Division of Diabetes Translation, Centers for Disease Control and Prevention, Atlanta, Georgia

**Figure:**
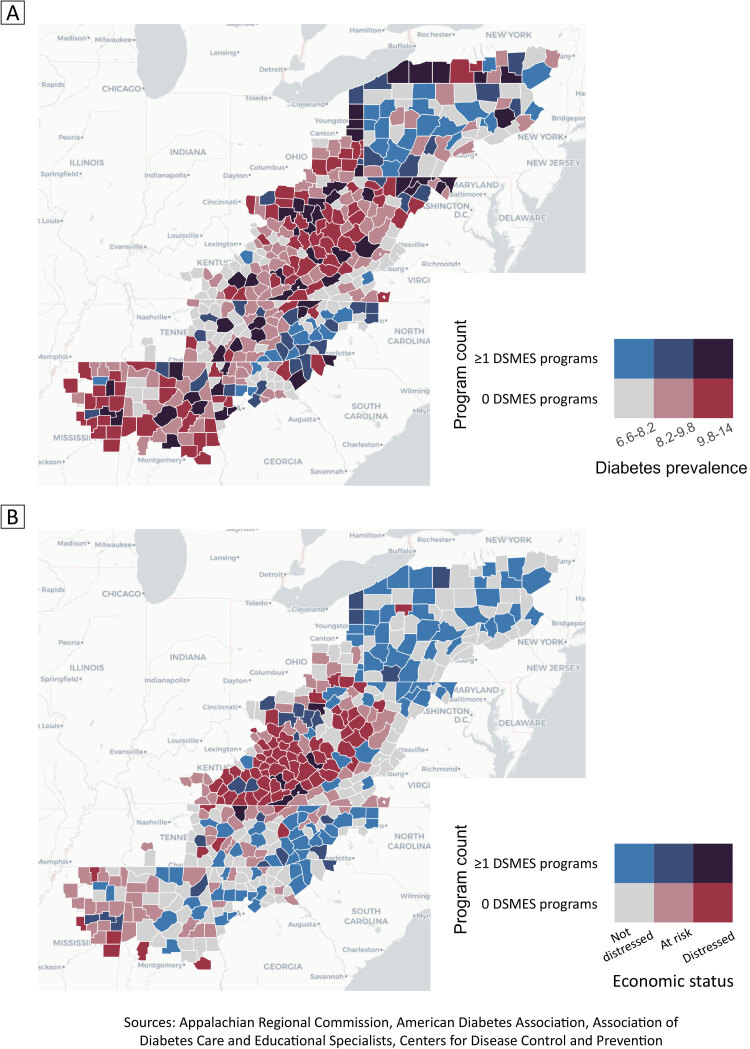
Bivariate choropleth maps of counties in the Appalachian region in 2021 showing the number of accredited or recognized programs offering diabetes self-management education and support (DSMES) services, by diabetes prevalence (Map A) and by county economic status of not distressed, at-risk, and distressed, as defined by the Appalachian Regional Commission (ARC) (Map B). Sources: ARC county data (14), American Diabetes Association or accredited by the Association of Diabetes Care and Education Specialists (DSMES program data); and diabetes prevalence (13).

## Introduction

Diabetes self-management education and support (DSMES) services provide information and skills for people to manage diabetes (1), as they reduce average hemoglobin A1c levels (2), improve quality of life (3), and improve the psychosocial aspects of managing diabetes (4). These services could empower people to set goals, develop self-care strategies, and adopt positive lifestyle changes, which contribute to improved diabetes management, enhanced overall health, lower health care costs, and reduced odds of hospitalization (5).

However, DSMES services are underused. Participation rates are 6.8% for people with private insurance in the first year of diagnosis, and those without insurance have 13% lower odds of participating (6,7). Barriers to use include limited access to services, distance from services, underdeveloped telehealth programs, lack of awareness about the benefits of DSMES, financial constraints, and limited health care provider referrals (8,9). Such barriers may be more pronounced in rural areas, such as much of the Appalachian region, which has higher rates of type 2 diabetes and worse health outcomes when compared with the US as a whole (10). Addressing these barriers and promoting use of DSMES services is crucial for comprehensive diabetes care and self-management.

This article focuses on economic equity in the Appalachian region and access to DSMES services in 2021. We describe the geographic association between diagnosed diabetes prevalence, economic distress, and number of programs recognized by the American Diabetes Association (ADA) or accredited by the Association of Diabetes Care and Education Specialists (ADCES) providing DSMES. Identifying areas with higher diabetes prevalence and a lower number of recognized programs in economically distressed counties may present an opportunity for enhanced clinical–community linkages.

## Data and Methods

We obtained addresses for programs recognized by the ADA or accredited by ADCES providing DSMES as of 2021. We geocoded these addresses using the prettymapr package (version 0.2.4) in R (version 4.2.1, R Foundation) to identify the county location for each program (11,12). For addresses that failed to geocode, we manually looked up the address to determine the county location. We summed the number of programs within each county and merged these data with county economic status data from the Appalachian Regional Commission (ARC). Data on diabetes prevalence in Appalachian counties were provided by the Centers for Disease Control and Prevention US Diabetes Surveillance System (13).

We used economic status and distressed areas data from the ARC for 2021 (14). Of the 423 counties that comprise the Appalachian region, 420 were included in the data set. We restricted our analysis to those 420 counties, of which 63.8% would be considered rural based on having a Rural–Urban Continuum code of 5 or more (15). The ARC groups counties into 5 categories: distressed, at-risk, transitional, competitive, and attainment. These categories are based on an index calculated for all counties nationally from 3 economic variables: 5-year estimated poverty rate (2014–2018), per capita market income (2018), and 3-year average unemployment rate (2016–2018). Distressed counties fall in the bottom 10% on this index, at-risk between 10% and 25%, transitional counties between 25% and 75%, competitive between 75% and 90%, and attainment counties are the top 10%. We collapsed counties initially categorized as transitional, competitive, and attainment into “not distressed.”

To describe the geographic association between diagnosed diabetes prevalence, economic distress, and number of accredited programs providing DSMES, we produced 2 maps. We also evaluated the association between program counts by county and economic status with a 2-part hurdle model due to excessive “zero” counts. This model uses a binomial model to first model absence versus presence (the “hurdle” component to be “cleared” before modeling the count) and a truncated negative binomial model to model counts for counties with 1 or more programs. We included economic status and diabetes prevalence as covariates and county population as an offset.

## Highlights

Of the 420 counties evaluated in the Appalachian region, 56.7% (n = 238) were not distressed, 24.8% (n = 104) were at-risk, and 18.6% (n = 78) were distressed. Of the 78 distressed counties, 48.7% (38 counties) were in Kentucky and 23.1% (18 counties) were in West Virginia. Diabetes prevalence in the Appalachian region was not significantly different between county economic status types. Prevalence ranged from 6.6% to 13.0%, with an average of 9.1% (95% CI, 9.0%–9.3%).

Of the 189 recognized or accredited programs providing DSMES in Appalachia, 154 (81.5%) were found in not-distressed counties, 28 (14.8%) in at-risk counties, and 7 (3.7%) in distressed counties. A total of 296 (70.5%) counties in Appalachia did not have any programs. Stratifying by economic distress category, we found that 60.0% (143 of 238) of not-distressed counties, 77.9% (81 of 104) of at-risk counties, and 92.3% (72 of 78) of distressed counties had no programs providing DSMES.

The binomial submodel from the hurdle model showed that distressed counties had 170.1% higher odds (adjusted odds ratio = 2.7; 95% CI, 1.1–6.7) of having no programs compared with not-distressed counties. Neither economic status nor diabetes prevalence in the truncated count model was significant.

## Actions

Economically distressed counties were least likely to have programs providing DSMES. Additionally, a substantial gap in programs providing DSMES existed between at-risk counties and not-distressed counties. These findings highlight a possible need for more equitable availability of DSMES services in the Appalachian region. Tailoring delivery modality, content, and frequency to the demographics and needs of the population may improve equitable access to these programs.

Despite the possibility of crossing county boundaries to access DSMES services, distance to available programs is a barrier to use (8,10). Because 70.5% of counties in the Appalachian region do not have DSMES programs, unless programs provide services in multiple counties, substantial lack of coverage is possible in this region. Future work could evaluate how use of DSMES services in Appalachia is affected by accredited program availability and other barriers to use, such as transportation availability, telehealth offerings, or cost. Public health organizations may facilitate increased clinical–community linkages with local clinics and community centers or help organize umbrella hub arrangements to increase the availability of DSMES services (16). Addressing disparities in availability of DSMES services could improve diabetes management outcomes and overall population health in Appalachia.
